# Radionuclide Removal from Aqueous Solutions Using Oxidized Carbon Fabrics

**DOI:** 10.3390/ma16237479

**Published:** 2023-12-02

**Authors:** Ioannis Ioannidis, Ioannis Pashalidis, Batuhan Mulla, Gkerman Kotanidis, Kyriacos Ioannou, Georgios Constantinides, Nikolaos Kostoglou, Claus Rebholz

**Affiliations:** 1 Department of Chemistry, University of Cyprus, P.O. Box 20537, Nicosia 1678, Cyprus; paschalidis.ioannis@ucy.ac.cy; 2 Department of Mechanical and Manufacturing Engineering, University of Cyprus, 1 Panepistimiou Avenue, Nicosia 2109, Cyprus; mulla.batuhan@ucy.ac.cy (B.M.); kotanidis.gkerman@ucy.ac.cy (G.K.); ioannou.kyriacos@ucy.ac.cy (K.I.); 3 Department of Mechanical Engineering and Materials Science and Engineering, Cyprus University of Technology, Lemesos 3036, Cyprus; g.constantinides@cut.ac.cy; 4 Department of Materials Science, Montanuniversität Leoben, Franz Josef-Strasse 18, 8700 Leoben, Austria; nikolaos.kostoglou@unileoben.ac.at

**Keywords:** U-232, Am-241, radioactivity, radionuclide removal, oxidized carbon fabrics, water treatment

## Abstract

The adsorption of actinide ions (Am(III) and U(VI)) from aqueous solutions using pristine and oxidized carbon fabrics was investigated by means of batch experiments at different pH values (pH 4, 7 and 9) and temperatures (25, 35 and 45 °C) under ambient atmospheric conditions. The experimental results indicated that both the pH and the fabric texture affected the adsorption rate and the relative removal efficiency, which was 70% and 100% for Am(III) and U(VI), respectively. The K_d_ (L/kg) values for U(VI) were generally found to be higher (2 < log_10_(K_d_)< 3) than the corresponding values for Am(III) adsorption (1.5 < log_10_(K_d_) < 2). The data obtained from the experiments regarding the temperature effect implied that the relative adsorption for both actinides increases with temperature and that adsorption is an endothermic and entropy-driven reaction. The application of the fabrics to remove the two actinides from contaminated seawater samples showed that both the relative removal efficiency and the K_d_ values decreased significantly due to the presence of competitive cations (e.g., Ca^2+^ and Fe^3+^) and complexing anions (CO_3_^2−^) in the respective waters. Nevertheless, the removal efficiency was still remarkable (50% and 90% for Am(III) and U(VI), respectively), demonstrating that these materials could be attractive candidates for the treatment of radionuclide/actinide-contaminated waters.

## 1. Introduction

The removal of radionuclides from contaminated waters (e.g., wastewater) prior to their release in the ocean or other environmental receivers is obligatory to protect the environment [1]. The present study deals with the removal of two actinide elements, uranium and americium, by means of their isotopes U-232 and Am-241, respectively. Uranium is a naturally occurring element and is found in the form of different isotopes, which are all radioactive with half-lives varying between 1.59 × 10^5^ and 4.47 × 10^9^ years [2]. The most abundant uranium isotopes in nature are U-238 (99.3%) and U-235 (0.7%) [3]. The latter is a fissile radionuclide and is widely used as the fuel in nuclear power reactors and nuclear weapons [4,5]. On the other hand, americium is a man-made element which is formed when plutonium absorbs neutrons during nuclear power production or nuclear power tests [6,7]. The most common isotopes of americium are Am-241 and Am-243, with half-lives of 432.2 and 7350 years, respectively. Am-241 is widely used in household smoke detectors and lightning rods. Furthermore, Am-241 has also been used as an alpha- and gamma-particle source for medical and industrial applications [6,8]. It is obvious that, due to the improper disposal of smoke detectors or even lightning rods, americium may enter landfills and disperse into the environment.

Uranium, under ambient conditions, basically exists in its hexavalent form as a uranyl cation (UO_2_^2+^) and in environmental waters can undergo hydrolysis [6,9] and carbonate complexation [10,11], and can interact with inorganic and organic colloids and mineral/rock surfaces [12,13]. In seawater, the U(VI)-tricarbonato complex (UO_2_(CO_3_)_3_^4−^) is the prevailing U(VI) species and stabilizes uranium in the aqueous phase. Under anoxic/reducing conditions, the reduction of U(VI) to U(IV) is also possible [14,15]. The trivalent oxidation state is the predominant oxidation state of americium in aqueous solutions and its chemical behavior is similar to the chemical behavior of trivalent lanthanides (e.g., Eu(III)) [16,17].

There are many methods (e.g., reverse osmosis, precipitation) to remove pollutants, including radionuclides, from waste and process waters [18,19,20,21]. However, the low cost and ease of operation, as well as the wide spectrum of adsorbent materials available, make adsorption-based technologies very attractive regarding the removal of pollutants [22,23,24]. In this respect, lightweight nanoporous carbon-based materials, including activated carbons, have been suggested as a promising category of adsorbents for the removal of pollutants from water sources due to their large surface areas and pore volumes, tunable and hierarchical pore structures and high adsorption capacities [25]. However, most of the commercial and lab-synthesized carbons are usually produced as fine and volatile powders, which could lead to potential issues concerning health, handling and practicality [26,27,28]. Instead, carbons produced in a more compacted form, including monoliths and cloths, are more likely to be used as functional components in commercial water purification systems and large-scale water treatment applications. For example, activated carbon cloth materials have attracted increasing interest over the last decade not only for wastewater treatment applications [29,30,31,32], but also for capacitive deionization [33], gas storage [34,35,36,37,38] and gas separation [35,36,37,38,39]. Activated carbon fabrics are strongly adsorbent materials, with physical and chemical stability, low density and breathability [37,38,40,41,42,43,44]. Furthermore, using nanoengineering techniques on carbon textiles shows promise in achieving elevated levels of properties and functionality [40,41,42,43]. Surface functionalization with strong Lewis bases (e.g., carboxylic acids and phosphates) usually results in an increased affinity for metal ions, including lanthanide and actinide ions [44]. Moreover, carbon fabrics can be adapted easily to existing filtration and water treatment systems, and could be an alternative substitute in adsorption-based water treatment technologies.

Hence, in this study, activated carbon fiber fabrics were used as flexible, robust and self-standing/binder-free adsorption-based filters for the removal of radionuclides (e.g., U-232 and Am-241) from laboratory solutions and seawater samples. To the best of our knowledge, this is the first study on the removal of radionuclides using activated carbon fabrics and their oxidized analogues, which are a special type of adsorbent with very attractive properties. The selected carbon fabrics demonstrate different macroscopic fibrous morphologies (i.e., woven, non-woven and felt) and pore structure characteristics (i.e., surface areas, pore volumes and pore sizes), as determined using scanning electron microscopy (SEM) and N_2_ adsorption tests. To move a step further, the same carbon fabrics were also controllably oxidized using chemical means with the aim to introduce surface oxygen-based functionalities, which could act beneficially for the removal of pollutants from water, as shown in a previous study [11,44,45]. Recently, Zulfiqar et al. reported a surface-functionalized carbon cloth that showed efficient separation of various types of oil–water mixtures under neutral, basic and acidic conditions [46]. Fourier-transform infrared (FTIR) and X-ray photoelectron spectroscopy (XPS) were also employed to investigate the differences in the surface chemistry and elemental composition between the pristine and oxidized carbon fabrics.

## 2. Materials and Methods

### 2.1. Materials

The tracer solutions employed in this study were Am-241 (North America Scientific Inc., Los Angeles, CA, USA) and U-232 (National Physical Laboratory, Teddington, UK), possessing activity concentrations of 12.05 and 4.923 kBq/g, respectively. These tracer standard solutions were utilized to create reference and test solutions, initially containing 25 Bq/L for each radionuclide, which equated to approximately 0.1 pmol/L for both Am-241 and U-232.

### 2.2. Methods

#### 2.2.1. Carbon Fabric Oxidation and Characterization

The three activated carbon fabrics used in this study (denoted hereafter as woven, non-woven and felt) were provided by Evertech Envisafe Ecology (Keelung City, Taiwan) and were controllably oxidized in the lab with concentrated HNO_3_ (8 M). A detailed description of the oxidation procedure of the fabrics is provided in a previous study [47]. It has been shown earlier that surface oxidation and formation of oxygen containing moieties such as carboxylic groups promote the interaction between metal ions and the biochar surface [45]. The three oxidized carbon fabrics are denoted hereafter as ox-woven, ox-non-woven and ox-felt, respectively.

SEM images were collected with an FEI Quanta 200 microscope (FEI, Hillsboro, OR, USA) using a 20 kV acceleration voltage and a working distance of approximately 10 mm. Prior to SEM analysis, a thin (a few nm) film of Au was deposited using sputter coating to improve surface conductivity and avoid potential charging effects during imaging. Images were collected at magnifications in the range of ×100 to ×20,000 in order to study their microstructural characteristics.

Surface area and pore structure analysis was performed by means of N_2_ adsorption/desorption at −196 °C using a manometric gas sorption analyzer (Anton-Paar QuantaTec Autosorb iQ_3_; Anton-Paar QuantaTec, Boynton Beach, FL, USA). He and N_2_ gases of ultra-high purity (99.999%) were employed for void volume calculations and gas sorption analysis, respectively. Prior to the tests, samples of ~40 mg were degassed under high vacuum (10^−6^ mbar) at 250 °C for 24 h to remove physisorbed water from their surface and make the pore structure more accessible. The specific surface area (SSA) was calculated using the multi-point Brunauer–Emmet–Teller (BET method), following the BET consistency criteria of the International Standard Organization (ISO 9277:2022 [48]). The specific pore volume (SPV) was calculated using the single-point Gurvich rule at relative pressures close to unity. The average pore width (W) was estimated using the ratio 2·SPV/SSA, upon assuming an infinitely extended slit-like pore system. XPS studies were carried out using a Thermo Scientific Theta Probe spectrometer (Waltham, MA, USA) equipped with a monochromated Al Kα radiation source (hv = 1486.6 eV) using an X-ray spot of ~400 mm in radius. Survey spectra were acquired using a pass energy of 300 eV, while a high-resolution core level spectrum for the C1s component was acquired with a pass energy of 50 eV. All spectra were charge referenced against the C1s peak at 284.5 eV (sp^2^ hybridized carbon) to correct for charging effects during acquisition. Quantitative chemical compositions were determined from the high-resolution core level spectra, using instrument-modified Wagner sensitivity factors, following the removal of a non-linear Shirley background. FTIR spectra were recorded by using a FTIR spectrometer 8900, Shimadzu (Tokyo, Japan).

#### 2.2.2. Adsorption Experiments

The sorption experiments were performed in 20 mL polyethylene (PE) vials under ambient conditions at 23 ± 2 °C. The experiments were performed in artificial solutions using deionized water at different pH values (4, 7 and 9) and sea water. The seawater samples corresponded to surface waters and were obtained from a coastal area in the southern part of the island of Cyprus (Larnaca district). The analysis of U-232 and Am-241 was carried out using an alpha-spectrometer (Canberra, Loches, France) after electrodeposition of uranium on stainless steel discs, as described elsewhere [49].

All sorption experiments were carried out by contacting 0.05 g of oxidized fabrics in artificial radionuclide solution (U-232 and Am-241) with a total volume of 10 mL. The contents of the flasks were stirred in a linear stirrer (SK-R1807, DLAB, Beijing, China) at a constant stirring rate (45 min^−1^). At specified time intervals, a small amount (50 µL) was withdrawn from the solution and electrodeposited to determine the adsorbed activity of the radionuclides. The radionuclide concentration in the solution was calculated with an alpha spectrometer. Spectrometer calibration was performed using a standard reference solution and a calibration source (1.02 Bq/mL U-232 standard reference solution and 6.6 Bq (total) U-238/234, Pu-239, Am-241 mixed standard on planchet by Eckert & Ziegler).

The trace amounts of uranium and americium isotopes used were extremely low. Relative to the initial concentration of radionuclides, surface binding sites (B) were expected to be in large excess. Therefore, to express the mass action of radiometal adsorption, the following distribution coefficient K_d_ can be used:

K_d_ = C_ads_/C_aq_ (L/Kg) (1)

C_ads_ (Bq/g) = the amount of radionuclide adsorbed by the fabrics;

C_aq_ (Bq/L) = the radionuclide concentration in solution at equilibrium.

The amount of radionuclides (U-232 and Am-241) adsorbed by the fabrics was calculated from the total amount of radionuclides adsorbed minus the relative amount of radionucleotides adsorbed by the plastic walls of the flask. The amount of radionucleotides adsorbed from the walls of the flask is significant and must be considered when adsorption experiments are performed using extremely low concentration levels. The percent adsorbed was calculated as the percent adsorbed amount of radioisotope in the test solution relative to the amount of radioisotope in the reference solution. All experiments were conducted twice and average values were employed for both data analysis and the creation of graphical representations.

## 3. Results and Discussion

### 3.1. Carbon Fabric Characterization

Both categories of pristine and oxidized carbon fabrics were characterized for their pore structure, morphology, surface chemistry and elemental composition using N_2_ adsorption, SEM, FTIR and XPS, respectively. Table 1 shows the pore structure properties, including the specific surface area, specific pore volume and average pore width, as derived from the analysis of N_2_ adsorption data using the BET method, the Gurvich rule and a combination of both, respectively. 

For the pristine samples, non-woven samples exhibited the highest surface area and pore volume among all samples, followed by very comparable values between the woven and felt samples. The average pore widths for all pristine samples seemed to range between 0.8 and 1 nm, indicating microporosity (i.e., pore sizes below 2 nm) according to the IUPAC nanopore classification [50]. Upon oxidation, all samples showed a significant decrease in their surface areas and pore volumes along with a slight increase in their average pore widths. More specifically, the surface area and pore volume values dropped by 71%, 50% and 56% and by 63%, 38% and 49% for ox-non-woven, ox-woven and ox-felt, respectively, compared to their non-oxidized counterparts. The average pore sizes of the oxidized samples shifted to higher values (i.e., between 1 and 1.2 nm), remaining, however, in the microporosity range. Generally, the non-woven fabrics presented a higher surface area because of their loose texture, which enabled a higher accessibility to the fabric pores. On the other hand, oxidation results in lower surface area, most probably because some of the micropores are “destroyed” during this process, thus leading to larger pore sizes. In addition, the formation of oxygen-containing moieties (e.g., carboxylic groups) makes the surface more hydrophilic and some of the micropores might be “blocked” and therefore cannot be accessed properly.

**Figure 1:**
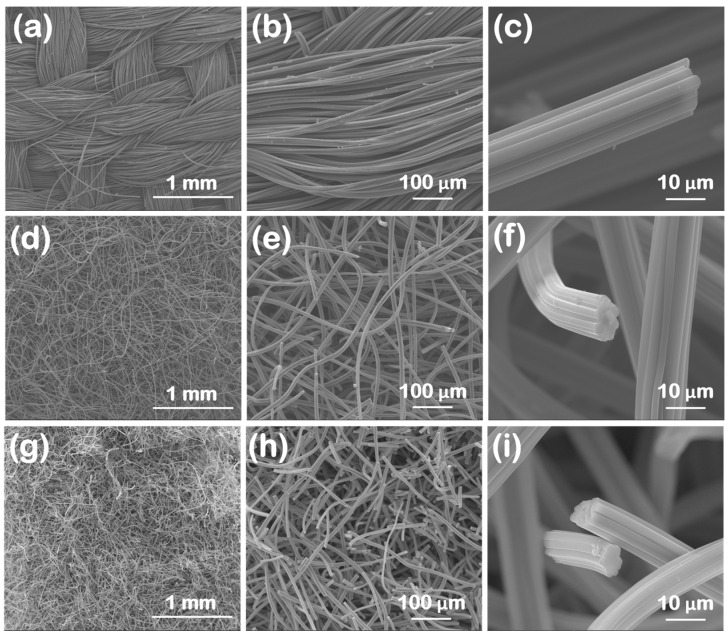
SEM images at different magnifications of the three oxidized carbon fabrics after oxidation with boiling 8 M HNO_3_. (woven (**a**–**c**), non-woven (**d**–**f**) and felt (**g**–**i**)).

SEM images for the oxidized carbon fabrics at three different magnifications, covering three orders of magnitude in length (i.e., from mm to μm), are illustrated in Figure 1. The macroscopic morphological features are highlighted in Figure 1a,d,g. The ox-woven sample was knitted from yarns that form a continuous fabric (Figure 1a), with each yarn spanning several hundreds of microns in width (i.e., up to ~400 μm) and consisting of tens of individual carbon fibers (Figure 1b). Instead, the ox-non-woven (Figure 1d,e) and ox-felt (Figure 1g,h) samples seemed to exhibit a more random “spaghetti”-like distribution of carbon fibers. The SEM images at the highest magnification (Figure 1c,f,i) revealed that, for all three cases, the carbon fibers were internally dense (not hollow), showed a corrugated circumference and had diameters in the range of 10–15 μm. It should be noted that all samples maintained the fibrous morphology of their non-oxidized counterparts.

**Figure 2:**
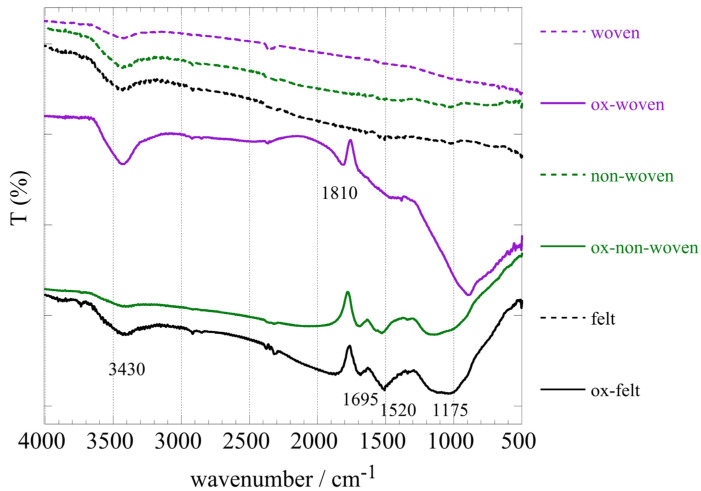
FTIR (KBr) spectra of the three oxidized carbon fabrics (woven, non-woven and felt) before and after oxidation with boiling 8 M HNO_3_.

Figure 2 shows the FT-IR spectra of the three fabric samples (woven, non-woven and felt) before and after oxidation. A comparison of the spectra before and after oxidation clearly indicated the successful modification of the fabric surface associated with the introduction of oxygen-containing moieties. Specifically, the peaks at 1695 cm^−1^, 1520 cm^−1^ and 1200 cm^−1^, which corresponded to –COO– and –C–O–C– vibrations, respectively, indicated the presence of carbonyl and carboxylic moieties on the fabric surface upon chemical oxidation [45]. It is notable that in the case of the woven fabric, the spectrum after oxidation differed significantly from the non-woven and felt ones, and the carbonyl peak at 1810 cm^−1^ was indicating rather carboxylic anhydrites. On the other hand, the peak at 3395 cm^−1^ could be ascribed to hydroxyl groups and water adsorbed on the material’s surface.

Table 2 reveals the elemental compositions of all fabric samples before and after oxidation, as derived from the XPS analysis. The pristine/non-oxidized samples demonstrated a high carbon purity (i.e., between 84 and 91 at.%), followed by oxygen (i.e., between 8 and 12 at.%) and much lower concentrations of nitrogen (i.e., between 0.5 and 3.5 at.%). Traces of phosphorus (P), silicon (Si) and sodium (Na) were also detected. The oxidized samples, however, showed a drop in their carbon content (i.e., between 72 and 77 at.%), along with a significant increase in their oxygen content (i.e., between 21 and 24 at.%) and a small increase in their nitrogen content (i.e., between 2 and 4 at.%). The aforementioned P, Si and Na traces were not detected anymore after the oxidation procedure. The XPS findings agreed well with the observations of the FTIR spectra, thus supporting the introduction of oxygen-containing moieties on the fibers.

### 3.2. Radionuclide Adsorption

**Figure 3:**
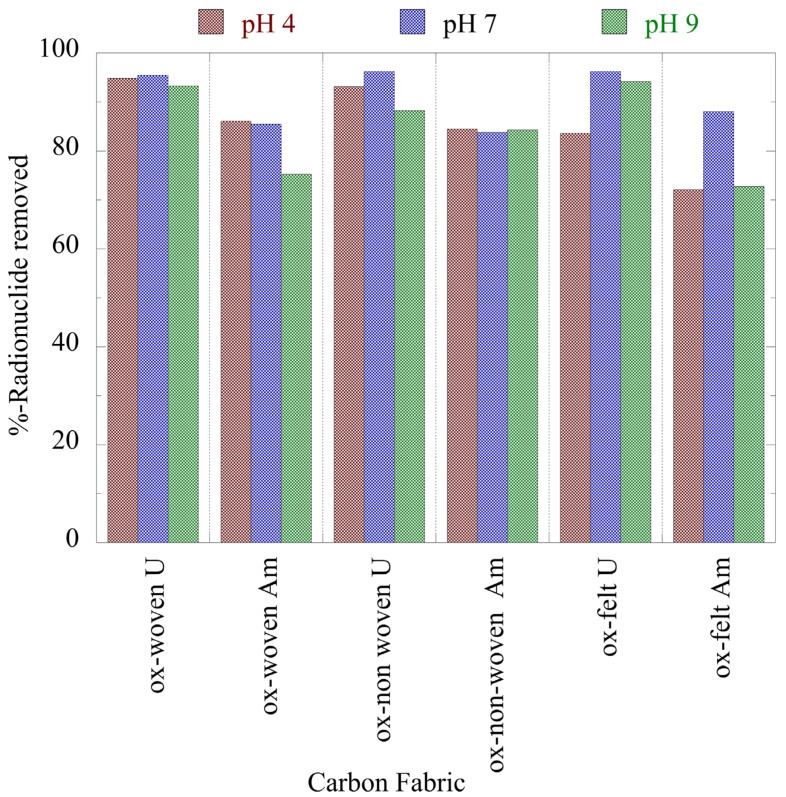
Relative removal of radionuclides (U-232 and Am-241) using the three oxidized carbon fabrics from aqueous solution in the acidic (pH 4), neutral (pH 7) and alkaline (pH 9) region. Experimental conditions: 0.5 Bq/mL of each radionuclide in 10 mL aqueous solution and ambient atmospheric conditions.

Figure 3 summarizes the relative removal efficiency determined for the adsorption of the two radionuclides (U-232 and Am-241) using the three oxidized carbon fabrics under acidic (pH 4), neutral (pH 7) and alkaline (pH 9) conditions. According to the collected data (Figure 3), uranium was more effectively adsorbed (>80%) by the fabrics than americium (>70%). Particularly for uranium, the highest removal efficiency was observed at a neutral pH, which was attributed to the partial protonation of the carboxylic groups in the acidic pH range and the stabilization of U(VI) due to the formation of UO_2_(CO_3_)_3_^4−^ in the alkaline pH region. Regarding americium, the three oxidized carbon fabrics presented different removal efficiencies in the different pH regions. The ox-non-woven fabric showed an almost similar removal efficiency independent of pH, while the removal efficiency of the ox-woven and the ox-felt adsorbents were significantly affected, particularly at pH 9. 

**Figure 4:**
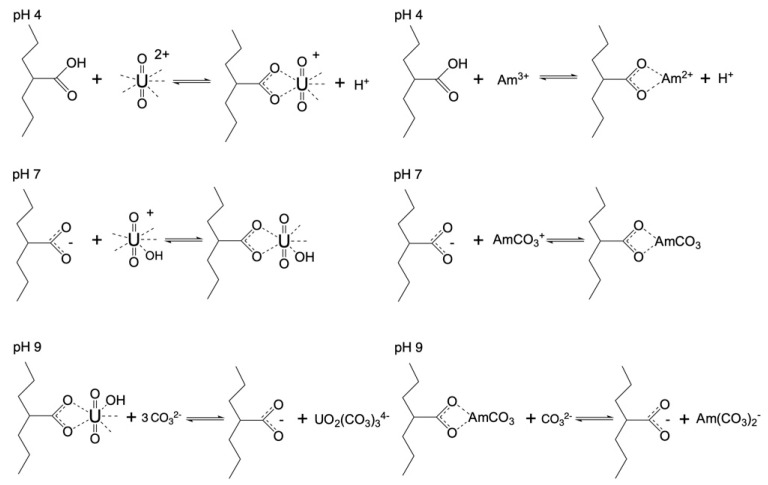
Schematic illustration of probable U(VI) and Am(III) binding by the oxidized carbon fabrics under acidic and alkaline pH conditions.

Possible reactions between the actinide ions (UO_2_^2+^ and Am^3+^) are schematically depicted in Figure 4. The proposed removal mechanism was based on previous studies in which depleted uranium [38] and Eu(III) were used an analogue for Am(III) [37]. In those studies, because of the low radioactivity of depleted uranium and the non-radioactive europium, the metal ion concentration was relatively high (up to mmol range) and the adsorption mechanism could be proven using spectroscopic measurements such as FTIR, XPS, etc. [37,38]. Furthermore, in the present study we expected a similar adsorption mechanism because U(VI) and Am(III) are hard Lewis acids and they will preferably interact with the hard Lewis bases, which, in the case of oxidized carbon fabrics, are the carboxylic moieties. Furthermore, because the mol-equivalent concentration of the carboxylic groups on the adsorbents surface was several orders of magnitude higher than the metal ion (radionuclide) concentration, the binding of the radionuclides by the carboxylic surface groups was expected to be the predominant adsorption mechanism. Moreover, in the near-neutral and alkaline pH region, hydrolysis and carbonate complexation of the actinide ions becomes predominant, resulting in the formation of ternary surface complexes at pH 7 and at pH 9 in the stabilization of the actinide ions as carbonate complexes, because the carbonate anion is the predominant ligand in the alkaline region under ambient conditions [10,11].

**Figure 5:**
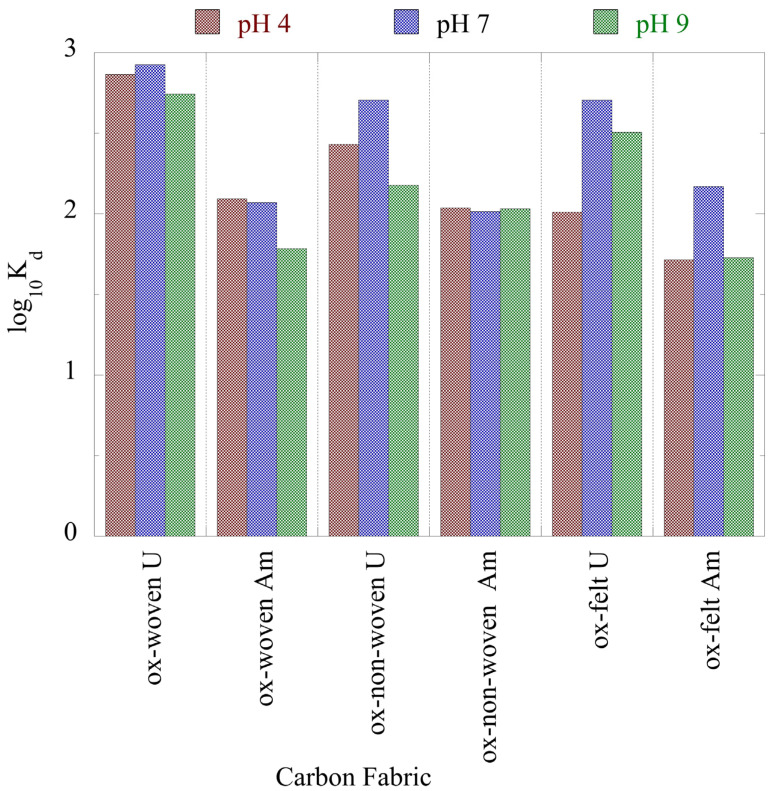
Adsorption efficiency (K_d_ values) associated with the adsorption of radionuclides (U-232 and Am-241) using three oxidized carbon fabrics from aqueous solution in the acidic (pH 4), neutral (pH 7) and alkaline (pH 9) region. Experimental conditions: 0.5 Bq/mL of each radionuclide in 10 mL aqueous solution and ambient atmospheric conditions.

The associated K_d_ values, which are summarized in Figure 5, show that the K_d_ values of U(VI) were almost one order of magnitude higher than the corresponding values of Am(III), indicating the higher affinity of the oxidized carbon fabrics for uranium. Nevertheless, the K_d_ values were lower than the K_d_ values determined for the adsorption of U(VI) and Am(III) using aerogel [51] and oxidized biochar fibbers [52], and higher than the K_d_ values determined for the adsorption of the respective radionuclides using microplastics [6,53,54]. These K_d_ values suggest that the oxidized carbon fabrics are excellent adsorbents for the removal of radionuclides from waters even at ultra-trace concentrations, and thus could be used in water treatment technologies for the efficient removal of radionuclides from radioactively contaminated waters.

#### 3.2.1. Adsorption Kinetics

**Figure 6:**
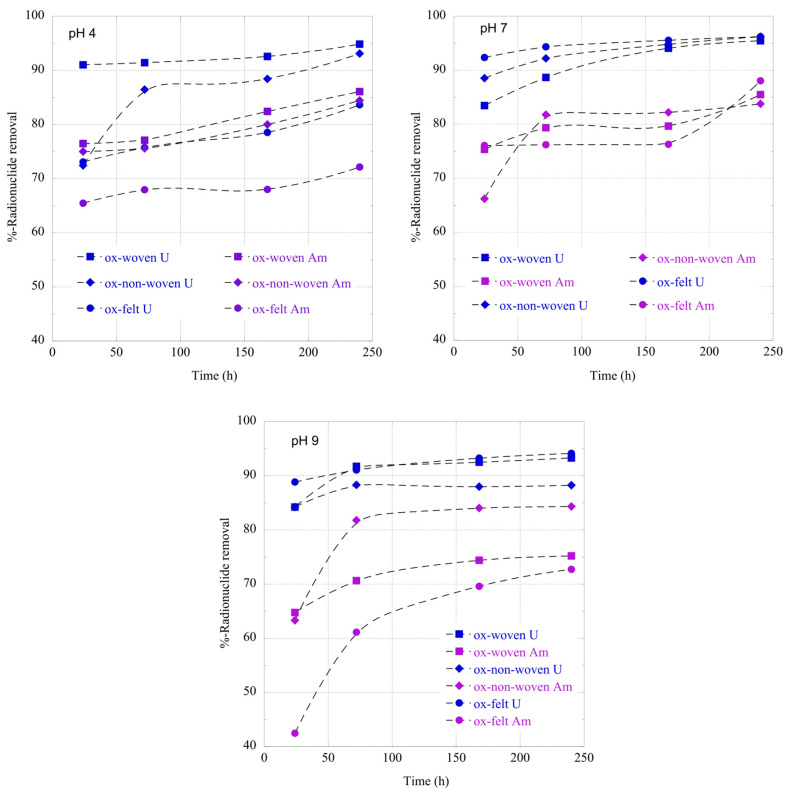
Relative removal of radionuclides (U-232 and Am-241) using the three oxidized carbon fabrics from aqueous solution in the acidic (pH 4), neutral (pH 7) and alkaline (pH 9) region as a function of time. Experimental conditions: 0.5 Bq/mL of each radionuclide in 10 mL aqueous solution and ambient atmospheric conditions.

The adsorption of radionuclides using the carbon fabrics is a relatively slow process and it takes approximately 10 days until a state of equilibrium is reached (Figure 6). This is common for adsorption studies performed at ultra-trace radionuclide concentrations because the adsorption kinetics are limited by the radionuclide diffusion from the solution to the adsorbent’s surface [23]. Generally, U(VI) is adsorbed faster than Am(III), most probably due to the higher affinity of the surface active moieties (e.g., carboxylic groups) for hexavalent uranium. Moreover, it is clear that the pH strongly affects the adsorption kinetics, because the speciation and hence the actinide species charge changes dramatically (see Figure 4), thus affecting the affinity of the fabric surface towards the different actinide species. Hence, the negative charge actinide species (e.g., carbonato species of U(VI) and Am(III)) in the alkaline region result in lower adsorption kinetics, particularly in the case of Am(CO_3_)_2_^−^.

#### 3.2.2. Adsorption Thermodynamics

**Figure 7:**
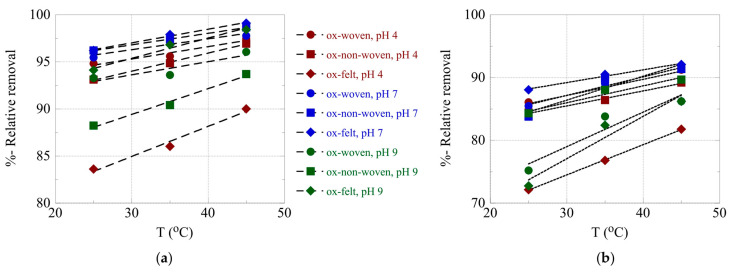
%-Relative removal as a function of T for the adsorption of (**a**) U(VI) and (**b**) Am(III) using the oxidized carbon fabrics. Experimental conditions: 0.5 Bq/mL of each radionuclide in 10 mL aqueous solution; the experiments were performed under different pH conditions (pH 4, 7 and 9) and at varied temperatures (25, 35, 45 °C).

**Figure 8:**
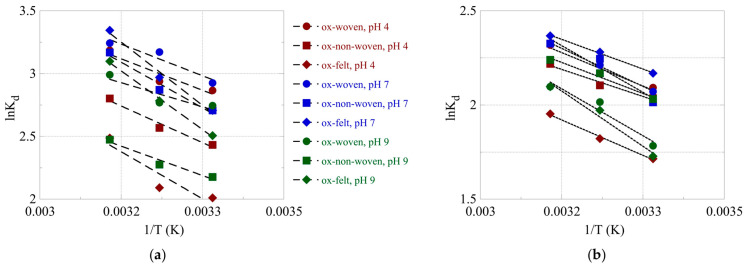
logK_ads_ as a function of 1/T for the adsorption of (**a**) U(VI) and (**b**) Am(III) using the oxidized carbon fabrics. Experimental conditions: 0.5 Bq/mL of each radionuclide in 10 mL aqueous solution; the experiments were performed under different pH conditions (pH 4, 7 and 9) and at varied temperatures (25, 35, 45 °C).

**Figure 9:**
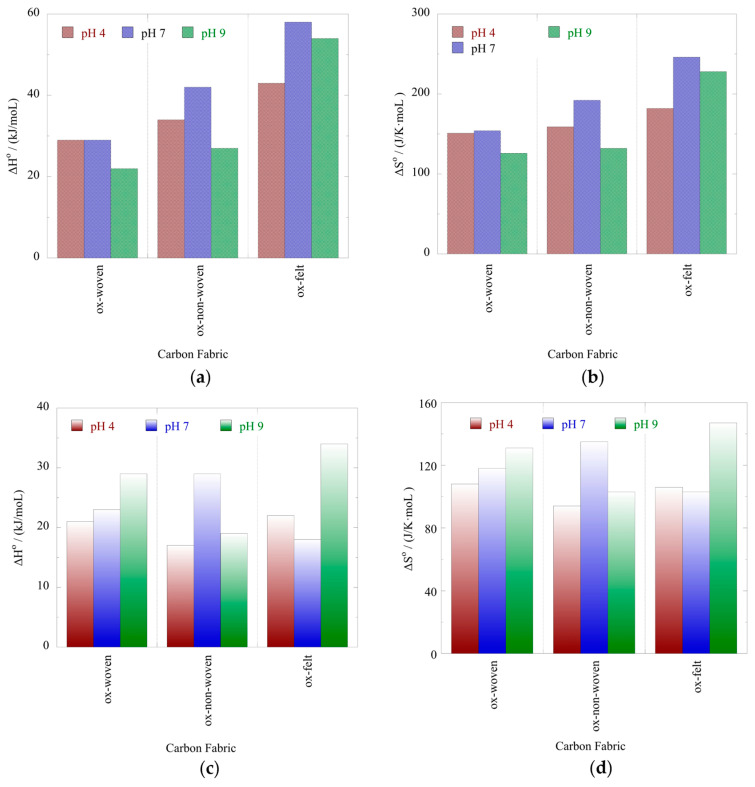
Standard enthalpy (ΔH°) (**a**,**c**) and standard entropy (ΔS°) (**b**,**d**) values for the adsorption of U(VI) and Am(III), respectively, using the oxidized carbon fabrics in de-ionized water solutions at varied pH values (pH 4, 7 and 9).

The effect of temperature on the relative adsorption and associated adsorption thermodynamic parameters (ΔG°, ΔH°, ΔS°) was evaluated using the experimental adsorption data obtained at three different temperatures. The %-relative adsorption and the lnK_ads_-1/T plots are graphically presented in Figure 7 and Figure 8, respectively. For all cases, increasing the temperature resulted in an increased relative adsorption, thus indicating that the radionuclide adsorption using the oxidized carbon fabrics is an endothermic, entropy-driven process. The values of the thermodynamic parameters (ΔH° and ΔS°) are summarized in Table 3 and graphically presented in Figure 9. This thermochemical behavior is generally observed for the adsorption of metal ions and other hard metal ions by surface-modified carbon-based materials and is associated with the formation of inner-sphere complexes between the surface carboxylate moieties and the radionuclide cations. Adsorption is an entropy-driven process, because upon adsorption and inner-sphere complex formation several water molecules are released from the hydration sphere of the radionuclide ion and hydrated surface carboxylates.

#### 3.2.3. Application to Seawater Samples

**Figure 10:**
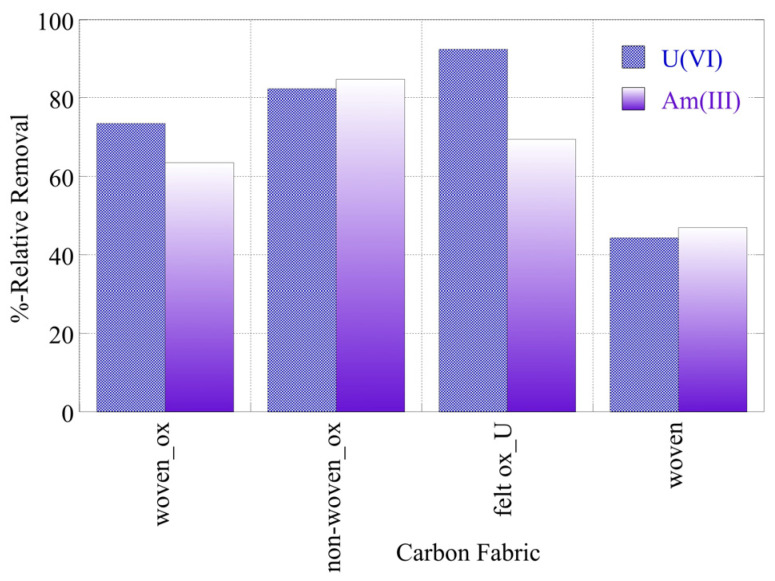
Relative removal of radionuclides (U-232 and Am-241) using three different oxidized carbon fabrics and the woven fabric from seawater solutions (pH 8.3). Experimental conditions: 0.5 Bq/mL of each radionuclide in 10 mL seawater solution and ambient atmospheric conditions.

**Figure 11:**
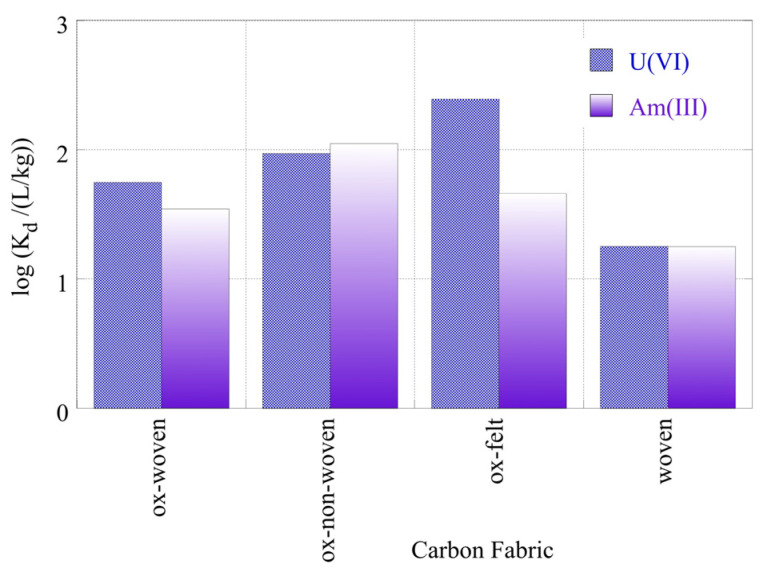
Adsorption efficiency (K_d_ values) associated with the adsorption of radionuclides (U-232 and Am-241) using three different oxidized carbon fabrics and the woven fabric from seawater solutions (pH 8.3). Experimental conditions: 0.5 Bq/mL of each radionuclide in 10 mL seawater solution and ambient atmospheric conditions.

The radionuclide (U-232 and Am-241) relative removal efficiency and adsorption efficiency (K_d_ values) using the three oxidized carbon fabrics and the three non-oxidized carbon fabrics in seawater (SW) samples are shown in Figure 10 and Figure 11, respectively. It was evident from Figure 9 that the relative removal efficiency and adsorption efficiency (K_d_ values) for oxidized carbon fabrics were reduced in relation to the corresponding values in DI water solutions, after restoration of the equilibrium. Specifically, the relative removal efficiency ranged from approximately 45 to 90% and the adsorption efficiency (log(K_d_)) values ranged approximately from 1.5 to 2.3, with the corresponding values in DI water at pH 9 ranging from 80 to 98% and from 1.8 to 2.9, respectively. This can be attributed to the presence of various cations (e.g., Ca^2+^ and Fe^3+^) and anions (e.g., CO_2_^3–^, SO_2_^4^) in the SW solution that compete with U(VI) and Am(III) ions, found in ultra-trace levels for adsorption on the surface of carbon fabrics. Moreover, the presence of carbonate anions stabilizes U(VI) and Am(III) in solution, forming carbonates such as UO_2_(CO_3_)_3_^4−^ and Am(CO_2_)_2_^−^.

This decrease in adsorptive capacity in ambient solutions such as SW was attributed to the adsorption of exceptionally low concentrations of radionuclides using various adsorbent materials such as microplastics [6] and aerogels [34]. The K_d_ values presented for oxidized carbon fabrics in SW were relatively higher than the corresponding values for U-232 and Am-241 adsorption with microplastics [6,52,53] and lower than those for aerogels [34].

The percentages for the relative removal and the K_d_ values for the adsorption of radionuclides decreased even further in the case of the non-oxidized (pristine) woven samples. This observation suggests the importance of the surface chemistry to the adsorbent material. Contrary to the non-oxidized woven samples, in the case of the oxidized woven samples the presence of oxo-groups (Figure 4) increased the adsorption capacity, as it allowed the development of interactions (e.g., dipole cation) between the oxygen on the surface and the various radionuclides (U (VI) and Am(III)). In addition, the texture of the carbon fabrics was also significant, because the loose fibers of the felt and non-woven fabrics resulted in higher adsorption efficiencies for the respective materials. The loose texture of these carbon fabrics was associated with a higher external surface area, which is of cardinal importance for metal ion adsorption. In a seawater solution,. due to the presence of competing cations, such as calcium and iron cations, surface active sites are predominantly occupied by the competing metal ions, which are at significantly higher concentrations. Hence, the higher external surface area of the felt and non-woven fabrics favors the adsorption of the actinide cations. 

## 4. Conclusions

U(VI) and Am(III) adsorption using oxidized carbon fabrics was investigated in DI water under various pH conditions (pH 4, 7 and 9) and SW (pH 8.3). The carbon fabrics presented a greater chemical affinity for U(VI) (relative removal efficiency > 80%) than Am(III) (relative removal efficiency > 70%) at all pH values studied in DI water. In fact, the K_d_ values of U(VI) were almost an order of magnitude greater than the corresponding values of Am(III). A higher adsorption capacity for U(VI) was observed at neutral pH while decreasing at slightly acidic and alkaline pH. This was due to the partial protonation of the carboxyl groups and the presence of anions (CO_2_^2−^) that stabilize the U(VI) in acidic and alkaline solutions, respectively. In the case of Am(III) adsorption using the oxidized non-woven fabric, the pH did not seem to significantly affect the adsorption. On the contrary, in the case of oxidized woven and felt fabrics, the adsorption of Am(III) was significantly affected by pH, especially at pH 9. Regarding the experiments carried out in SW, the adsorption capacity for both radionuclides was reduced due to the competitive behavior of the various cations (e.g., Ca^2+^ and Fe^3+^) and anions (e.g., CO_2_^3–^, SO_2_^4−^) present in the solution. The adsorption of the two radionuclides is a relatively slow process, with U(VI) being absorbed faster than Am(III). On the other hand, adsorption is favored by an increased temperature, as in all cases the relative adsorption increased with increasing temperatures, indicating that the adsorption of U(VI) and Am(III) is an endothermic and entropy-driven process.

## Figures and Tables

**Table 1:** Pore structure properties for the pristine and oxidized carbon fabrics derived from N_2_ adsorption data recorded at 77 K.

Material	S_BET_ [m^2^/g]	V_Gurvich_ [nm]	W [nm]
Woven	1119	0.47	0.84
Ox-woven	560	0.29	1.03
Non-woven	1845	0.86	0.93
Ox-non-woven	532	0.32	1.20
Felt	1080	0.49	0.90
Ox-felt	475	0.25	1.05

S_BET_: Brunauer–Emmet–Teller (BET) surface area, V_Gurvich_: total pore volume at P/P_0_ ~0.95 for pores less than 50 nm in width calculated using the single-point Gurvich rule, W: average pore width calculated using the ratio of 2·(V_Gurvich_)/(S_BET_) assuming a slit pore model.

**Table 2:** Elemental compositions of pristine and oxidized carbon fabrics based on XPS data.

Material	Element (at.%)					
	C	N	Na	O	P	Si
Woven	83.5	3.6	0.1	11.7	0.9	0.2
Ox-woven	72.3	3.8	--	23.8	0.2	--
Non-woven	90.5	0.5	0.5	7.5	0.5	0.6
Ox-non-woven	75.6	1.6	0.1	22.6	--	--
Felt	89.6	1.3	0.3	8	0.5	0.2
Ox-felt	76.8	2.1	--	21.1	--	--

**Table 3:** Thermodynamic parameters obtained from the evaluation of the adsorption data at different temperatures.

Material/pH	U(VI)		Am(III)	
	ΔH° (kJ/moL)	ΔS° (J/K·moL)	ΔH° (kJ/moL)	ΔS° (J/K·moL)
Ox-woven/4	29	151	21	108
Ox-non-woven/4	34	159	17	94
Ox-felt/4	43	182	22	106
Ox-woven/7	29	154	23	118
Ox-non-woven/7	42	192	29	135
Ox-felt/7	58	246	18	103
Ox-woven/9	22	126	29	131
Ox-non-woven/9	27	132	19	103
Ox-felt/9	54	228	34	147

## Data Availability

The data presented in this study are available on request from the corresponding authors.
